# Identification of an individualized RNA binding protein‐based prognostic signature for diffuse large B‐cell lymphoma

**DOI:** 10.1002/cam4.3859

**Published:** 2021-03-21

**Authors:** Yongzhi Xie, Ximei Luo, Haiqing He, Tao Pan, Yizi He

**Affiliations:** ^1^ Department of Neurology The Third Xiangya Hospital Central South University Changsha China; ^2^ School of Computer Science and Technology Harbin Institute of Technology Harbin China; ^3^ Department of Urology Second Xiangya Hospital Central South University Changsha China; ^4^ Department of Lymphoma & Hematology The Affiliated Tumor Hospital of Xiangya Medical School Central South University Changsha China

**Keywords:** diffuse large B‐cell lymphoma, GEO, prognosis, prognostic model, RNA‐binding proteins

## Abstract

RNA binding proteins (RBPs) are increasingly appreciated as being essential for normal hematopoiesis and have a critical role in the progression of hematological malignancies. However, their functional consequences and clinical significance in diffuse large B‐cell lymphoma (DLBCL) remain unknown. Here, we conducted a systematic analysis to identify RBP‐related genes affecting DLBCL prognosis based on the Gene Expression Omnibus database. By univariate and multivariate Cox proportional hazards regression (CPHR) methods, six RBPs‐related genes (*CMSS1*, *MAEL*, *THOC5*, *PSIP1*, *SNIP1*, and *ZCCHC7*) were identified closely related to the overall survival (OS) of DLBCL patients. The RBPs signature could efficiently distinguished low‐risk from high‐risk patients and could serve as an independent and reliable factor for predicting OS. Moreover, Gene Set Enrichment Analysis revealed 17 significantly enriched pathways between high‐ versus low‐risk group, including the regulation of autophagy, chronic myeloid leukemia, NOTCH signaling pathway, and B cell receptor signaling pathway. Then we developed an RBP‐based nomogram combining other clinical risk factors. The receiver operating characteristic curve analysis demonstrated high prognostic predictive efficiency of this model with the area under the curve values were 0.820 and 0.780, respectively, in the primary set and entire set. In summary, our RBP‐based model could be a novel prognostic predictor and had the potential for developing treatment targets for DLBCL.

## Graphical abstract

We found that six RNA binding proteins are significantly related to the overall survival of diffuse large B‐cell lymphoma (DLBCL) patients, which could potentially be served as prognostic biomarkers. Gene Set Enrichment Analysis provided insight into the underlying mechanisms in the occurrence and development of DLBCL, which laid the foundation for further basic studies.

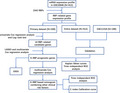

## 1 INTRODUCTION

Diffuse large B‐cell lymphoma (DLBCL) is an aggressive form of non‐Hodgkin lymphoma with variations in gene expression profiles and genetic alterations displaying the difference in clinical course and response to therapy.^1^ In the past two decades, rituximab plus CHOP (R‐CHOP) immunochemotherapy (rituximab plus cyclophosphamide, doxorubicin, vincristine, and prednisone) significantly extended the survival and increased the patient cure rate, with 50%–70% of patients being cured using this approach.^2^ However, the efficacy of salvage options for the refractory/relapse DLBCL patients is diminished. The ability to identify a very poor risk group with low probability of cure following R‐CHOP at diagnosis is a major unmet clinical need.^3^, ^4^

Gene expression profiling studies have demonstrated that distinct B‐cell differentiation stages are not only regulated by transcription factors but also affected by post‐transcriptional regulatory mechanisms, including RNA binding proteins (RBPs).^1^, ^5^ RNAs have been identified as oncogenic drivers and tumor suppressors in every major cancer type.^6^, ^7^, ^8^ RBPs modulate multiple cancer traits and regulate a multitude of cellular processes, including subcellular localization, translational repression and stability, and the control of cell proliferation.^8^, ^9^ Perturbations in RBP‐RNA networks activity have been causally associated with cancer development.^7^, ^10^ Because of its pleotropic effects, an increasing number of studies have point to the crucial role of RBPs in hematopoietic malignancies.^8^, ^11^, ^12^ However, whether RBPs can independently act as a biomarker that can predict the clinical outcomes of therapeutic strategies in DLBCL remains unknown. Meanwhile, a deeper understanding of the normal role of RBPs complex networks would provide a unique opportunity to unveil better interventions for the treatment of R/R DLBCL.

In this study, we extracted RNA sequencing and clinical data from the Gene Expression Omnibus (GEO) database and constructed a 6‐RBP‐based gene signature to predict the prognostic risk of DLBCL. Then, we developed a composite prognostic nomogram combining the RBP‐based prognostic signature with clinical risk factors, which demonstrated higher predictive efficiency than their separation. Overall, our data suggest that RBPs play pivotal roles in DLBCL and the RBP‐based model could be used as reliable prognostic predictors for DLBCL survival.

## 2 MATERIALS AND METHODS

### 2.1 Data acquisition

The public gene expression profiling and clinical data were available in the GEO database (https://www.ncbi.nlm.nih.gov/geo/). Two DLBCL related datasets GSE10846 and GSE11318, which used the GPL570 platform, were obtained in a normalized expression matrix file format. To clarify the association between gene expression signatures and overall survival (OS) of DLBCL patients, samples without accurate survival data were filtered out. Thus, DLBCL data for a total of 412 patients from the GSE10846 and 199 patients from the GSE11318 were included in this study. A total of 1542 RBP‐related genes had been reported previously and were analyzed in this study.^9^

### 2.2 Screening of OS‐related RBP in DLBCL patients

The matrix of 1542 RBP‐related gene expression was first extracted. The univariate Cox proportional hazards regression (CPHR) and the Kaplan–Meier (K–M) analysis were performed by “survival” R package. Genes with *p* ≤ 0.01 and with their expression logical consistency with the prognostic effects were considered as candidate prognostic RBP‐related genes.

### 2.3 Identification and assessment of the RBP‐related gene signature

To minimize the number of genes more closely related to OS, the least absolute shrinkage and selection operator (LASSO) Cox regression was performed by “glmnet” R package. Then, multivariate CPHR (stepwise model) analysis was applied by “survival” R package to construct a prognostic model. Prognostic risk score was calculated by following formula: risk score = Exp_1_*Coef_1_ + Exp_2_*Coef_2_ + Exp_3_*Coef_3_ + … Exp_n_*Coef*_n_*. Exp is the expression level of hub‐gene, and Coef is the regression coefficients of the corresponding gene derived from the multivariate CPHR. Median risk score was used as a cut‐off value to divided patients into low‐ and high‐risk groups.

### 2.4 Gene Set Enrichment Analysis

To explore the KEGG pathways that may be related to high‐ or low‐risk patients, we performed Gene Set Enrichment Analysis (GSEA) between the high‐ and low‐risk groups based on identified gene signature. GSEA_v4.1.0 (http://www.gsea‐msigdb.org/gsea/index.jsp) was used for analysis according to default parameters and c2.cp.kegg.v6.2.symbols.gmt was used as a reference gene list. The pathways with *p* < 0.05 and normalized enrichment score |NES| ≥ 1 were considered significant.

### 2.5 Construction and validation of the prognostic nomogram

We further performed univariate and multivariate CPHR analysis of conventional clinical risk factors and RBP‐related signature to identify independent prognostic factor of OS in the GSE10846 dataset. A prognostic nomogram combining gene signature and OS‐related clinical factors was established by “rms” R package. Calibration curves and concordance index(C‐index) were used to evaluate the abilities of the nomogram.

### 2.6 Statistical analysis

Kaplan–Meier survival curves and log‐rank test were performed by utilizing “survival” and “survminer” R package to demonstrated survival differences between the two groups. Time‐dependent receptor operating characteristic (ROC) analysis was performed by “survivalROC” R package to assess the accuracy of the prognostic model. *p* < 0.05 was indicated statistically significant.

## 3 RESULTS

### 3.1 Identification of OS‐related RBP

**FIGURE 1:**
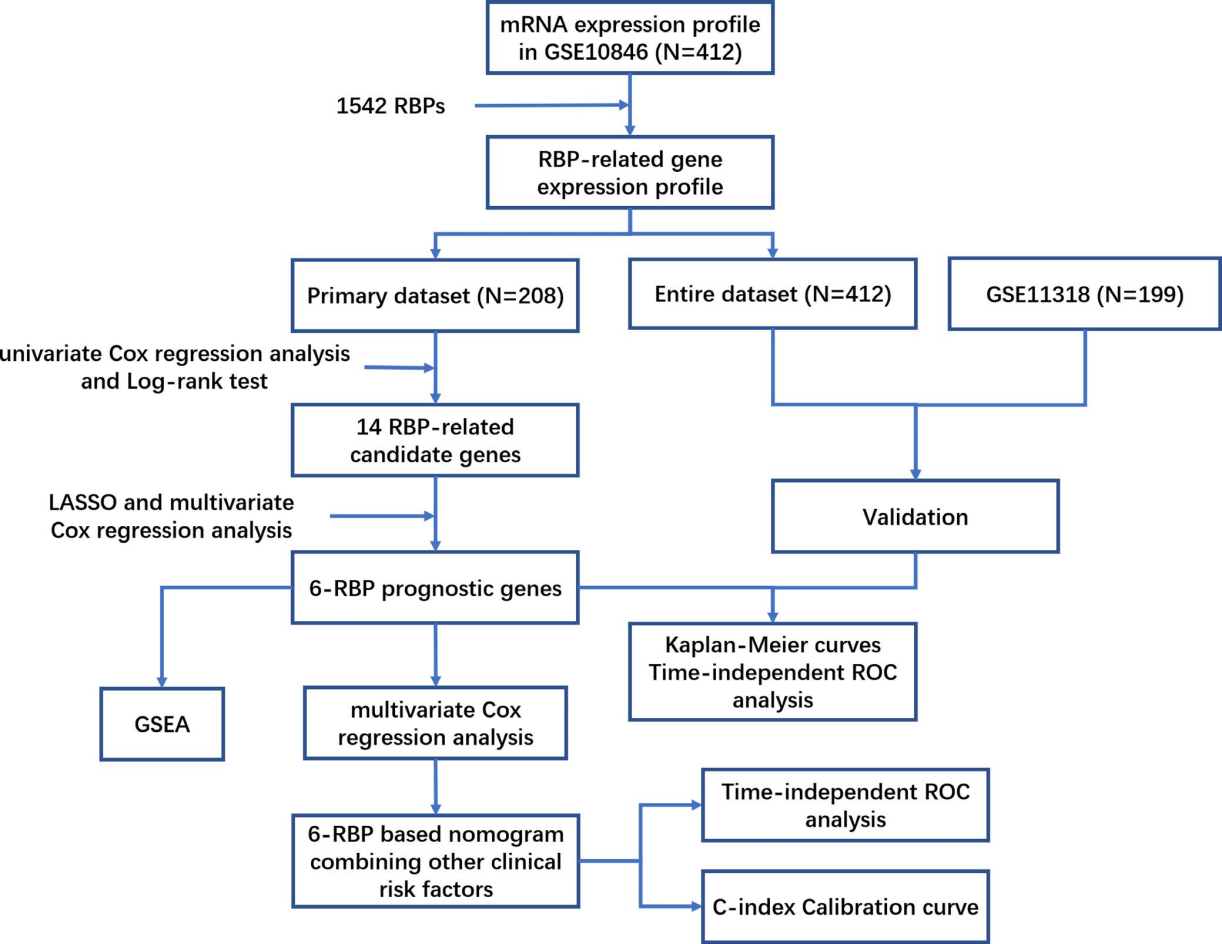
Flowchart of the present study

As the workflow of this study presented in Figure 1, we defined 412 patients from the GSE10846 as the “entire set,” 208 of them were randomly assigned as the “primary set.” The patients in the primary set were used as a training cohort, and the patients in the entire set and GSE11318 were used as validation cohort (Table 1).

**TABLE 1:** Baseline clinical features of diffuse large B‐cell lymphoma patients involved in this study

Features	GSE10846 dataset (*n* = 412)		GSE11318 dataset (*n* = 199)
	Primary set (*n* = 208)	Entire set (*n* = 412)	
Gender			
Male	108	222	109
Female	91	172	90
NA	9	18	
Age			
≤60	91	188	69
<60	117	224	93
NA			37
Subtype			
GCB	86	182	70
Non‐GCB	122	230	129
ECOG			
≤1	148	295	122
>1	51	93	39
NA	9	24	38
Stage			
I–II	89	188	75
III–IV	115	217	87
NA	4	7	37
Treatment			
CHOP	83	180	162
R‐CHOP	125	232	
NA			37

Abbreviations: CHOP, cyclophosphamide, doxorubicin, vincristine, and prednisone; ECGO, eastern cooperative oncology group score; GCB, germinal center B‐cell‐like; NA, not available; R‐CHOP, rituximab plus CHOP.

**FIGURE 2:**
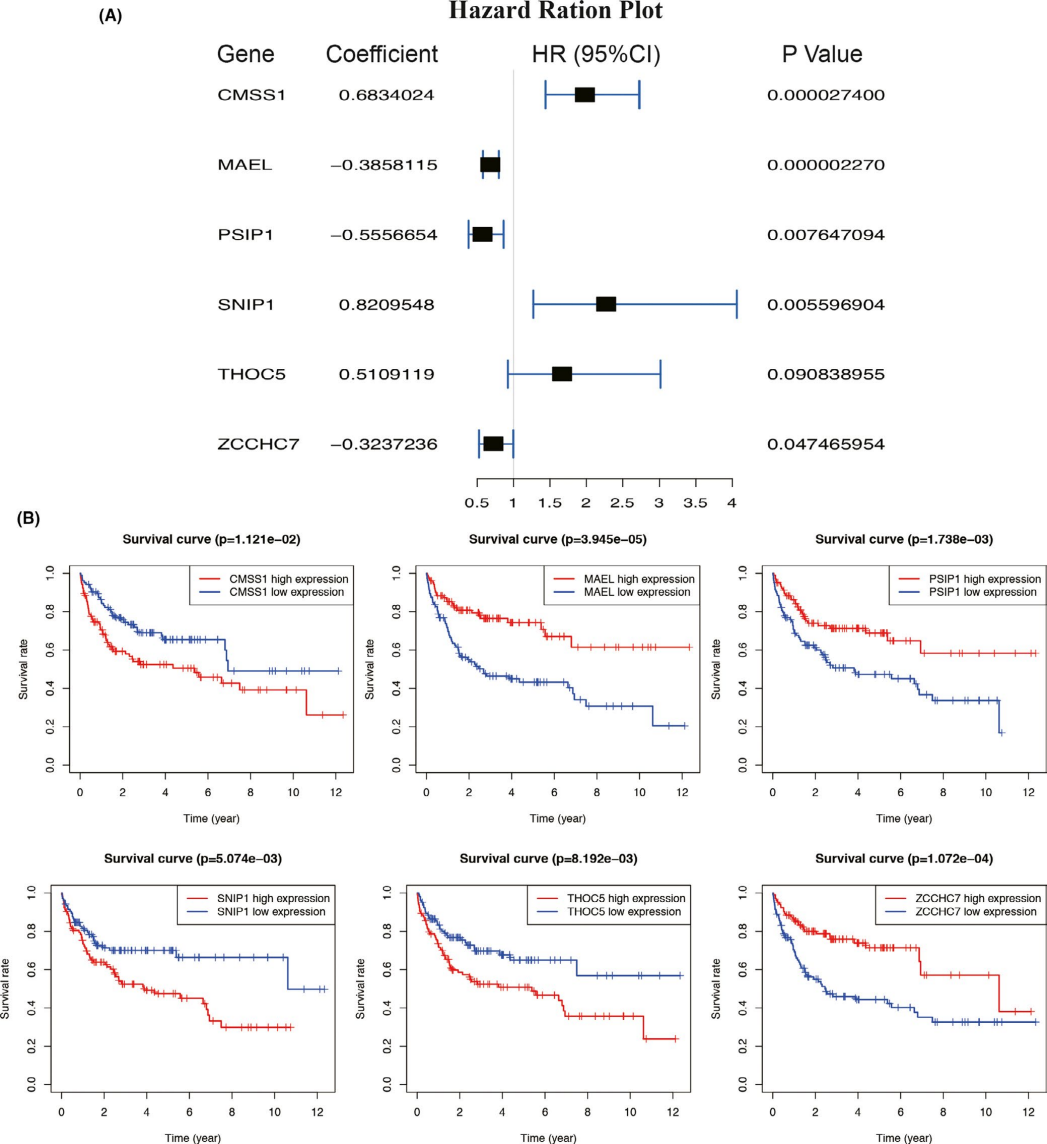
Identification of Six RNA binding protein (RBP)‐related genes associating with overall survival. (A) Forest plot of six prognostic RBPs in diffuse large B‐cell lymphoma based on the multivariate Cox regression analysis. (B) Kaplan–Meier analysis of six RBP‐related genes. CI, confidence interval. *p* < 0.05 was considered statistically significant

To identify OS‐related RBP in DLBCL patients, 1542 RBP‐related genes were subjected to univariate CPHR and the K–M survival analysis in the training set. We set the *p* ≤ 0.01 as the screening criteria, and 14 RBP‐related candidate genes were identified associating with OS (Figure S1A). We then performed a LASSO regression analysis (Figure S1B) and multivariate CPHR analysis (stepwise model) on these 14 genes to build a best‐fit prognostic signature. Six RBP‐related genes (*CMSS1*, *MAEL*, *THOC5*, *PSIP1*, *SNIP1*, and *ZCCHC7*) were finally selected to predict OS in DLBCL patients (Figure 2A). The K–M curves of six genes could clearly distinguish two groups (all *p* < 0.05), consistent with the results of Cox regression analysis (Figure 2B). The risk score of the prognostic signature was established as following formula: Risk score = (0.683 × Exp[CMSS1]) + (−0.386 × Exp[MAEL]) + (−0.556 × Exp[PSIP1]) + (0.821 × Exp[SNIP1]) + (0.511 × Exp[THOC5]) + (−0.324 × Exp[ZCCHC7]).

### 3.2 Assessment and validation of the six‐RBP prognostic signature

**FIGURE 3:**
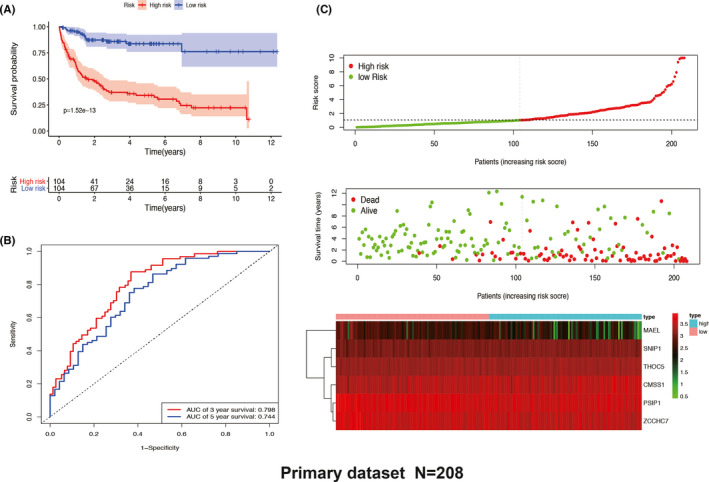
Six‐RNA binding protein (RBP) signature‐based risk score analysis of primary dataset in GSE10846. (A) Kaplan–Meier analysis of patients with high‐ and low‐risk score. (B) Time‐dependent receiver operating characteristic curve analysis for evaluating the 3‐ and 5‐year predictive performance based on the six‐RBP gene signature. (C) Six‐RBP signature‐based risk score distribution, overall survival status, and heatmap of gene expression. AUC, area under the curve

Based on the median risk score, DLBCL patients in the training set (primary set, *N* = 208) were divided into high‐ and low‐risk groups. The K–M survival analysis showed that the DLBCL patients with high‐risk score had a poor prognosis than those with low‐risk score (*p* = 1.52e−13) (Figure 3A). The time‐dependent ROC curves demonstrated that the area under the curve (AUC) of the six‐RBP signature reached 0.798 and 0.744 at 3 and 5 years, respectively (Figure 3B). The risk scores, survival status, and heatmap of six‐RBP expression are shown in Figure 3C.

**FIGURE 4:**
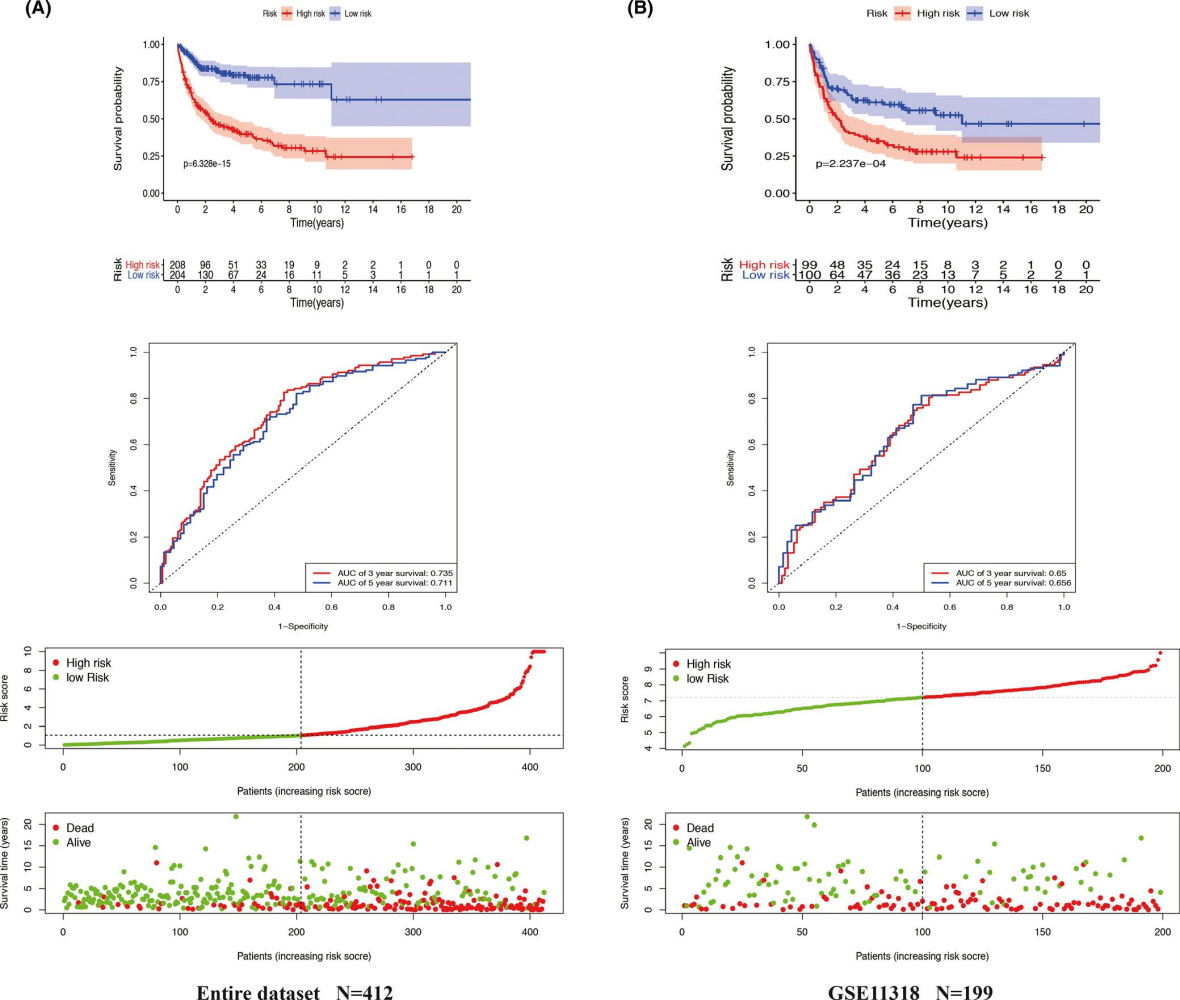
Validation of Six‐RNA binding protein (RBP) gene signature in the entire dataset of GSE10846 and in the GSE11318 dataset. The six‐RBP gene signature‐based Kaplan–Meier analysis for overall survival (OS), time‐dependent receiver operating characteristic curve analysis for predicting 3‐ and 5‐year OS, risk score distribution, and OS status analysis of patients in the entire dataset of GSE10846 (A) and in the GSE11318 dataset (B)

We further validated the six‐RBP prognostic signature in the entire set (*n* = 412) (Figure 4A) and GSE11318 dataset (*n* = 199) (Figure 4B). The distribution of risk score and survival status in the entire set and GSE11318 were consistent with the results showing above. The K–M survival analysis showed that high‐risk score associated with poor survival time in the primary set (*p* = 6.328e−15) and GSE11318 (*p* = 2.237e−04). The six‐RBP prognostic signature achieved the AUC of 0.735 at 3 years and 0.711 at 5 years in the entire set and achieved the AUC of 0.65 at 3 years and 0.656 at 5 years in the GSE11318 dataset.

Because rituximab‐based regimens are cost‐prohibitive for many patients, we then validated the six‐RBP signature based on different treatments (CHOP or R‐CHOP). The results showed that our established six‐RBP signature could also efficiently differentiate high‐ versus low‐risk patients (*p* = 1.485e−05 in CHOP; *p* = 7.594e−09 in R‐CHOP) and accurately predict OS (AUC = 0.707 in CHOP; AUC = 0.769 in R‐CHOP) (Figure S2).

### 3.3 Gene Set Enrichment Analysis

**FIGURE 5:**
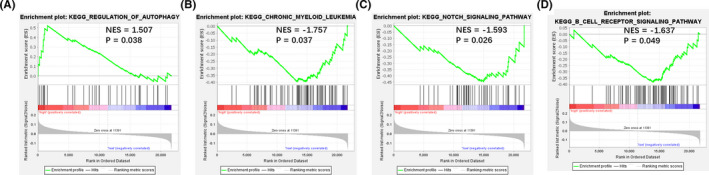
Gene set enrichment analysis (GSEA) between high‐ and low‐risk score groups in GSE10846. (A) Regulation of autophagy. (B) Chronic myeloid leukemia. (C) NOTCH signaling pathway. (D) B cell receptor signaling pathway

To explore the potential influence of six‐RBP signature on the expression profile of DLBCL, GSEA analysis was applied in the GSE10846 cohort (high‐ vs. low‐risk group). A total of 17 KEGG pathways were identified (Table S1), among which the regulation of autophagy, chronic myeloid leukemia (CML), NOTCH signaling pathway, and B cell receptor signaling pathway are close related to lymphoma or hematological malignancies (Figure 5).

### 3.4 Univariate and multivariate CPHR of six‐RBP prognostic signature

To determine whether our prognostic signature act as an independent prognostic factor, univariate and multivariate CPHR analyses were performed in primary and entire sets. The risk score of six‐RBP and clinical factors, including gender, age, DLBCL subtype (germinal center B‐cell‐like [GCB] or non‐GCB), eastern cooperative oncology group score (ECOG), and stage, were used as covariates. Results showed that the six‐RBP signature, age, DLBCL subtype, ECOG score, and stage were significantly associated with OS (all *p* < 0.05), confirming that the six‐RBP signature could be an independent prognostic predictor of DLBCL patients (Table 2).

**TABLE 2:** Univariate and multivariate Cox analysis of six‐RNA binding protein signature and clinical factors in the GSE10846 dataset

Feature	Univariate COX		Multivariate COX	
	HR (95% CI)	*p*	HR (95% CI)	*p*
Entire dataset				
Gender (male vs. female)	0.970 (0.707–1.331)	0.849	0.961 (0.705–1.311)	0.801
Age (>60 vs. ≤60 years)	1.733 (1.233–2.436)	0.002	2.019 (1.446–2.820)	<0.001
Subtype (non‐GCB vs. GCB)	1.527 (1.060–2.198)	0.023	2.384 (1.695–3.352)	<0.001
ECOG (>1 vs. ≤1)	1.885 (1.339–2.654)	<0.001	2.874 (2.076–3.979)	<0.001
Stage (III–IV vs. I–II)	1.525 (1.084–2.146)	0.015	1.875 (1.356–2.592)	<0.001
Risk score (high vs. low)	2.863 (1.933–4.240)	<0.001	4.008 (2.774–5.790)	<0.001
Primary dataset				
Gender (male vs. female)	0.898 (0.572–1.410)	0.641	0.867 (0.566–1.328)	0.512
Age (>60 vs. ≤60 years)	1.540 (0.942–2.517)	0.085	1.921 (1.210–3.048)	0.006
Subtype (non‐GCB vs. GCB)	1.476 (0.893–2.440)	0.129	2.445 (1.504–3.977)	<0.001
ECOG (>1 vs. ≤1)	1.886 (1.182–3.004)	0.008	3.127 (2.015–4.852)	<0.001
Stage (III–IV vs. I–II)	1.841 (1.128–3.004)	0.015	1.928 (1.223–3.039)	0.005
Risk score (high vs. low)	4.578 (2.564–8.175)	<0.001	6.307 (3.604–11.035)	<0.001

Abbreviations: 95% CI, 95% confidence interval; ECGO, eastern cooperative oncology group score; GCB, germinal center B‐cell‐like; HR, hazard ratio.

### 3.5 Construction of the prognostic nomogram

**FIGURE 6:**
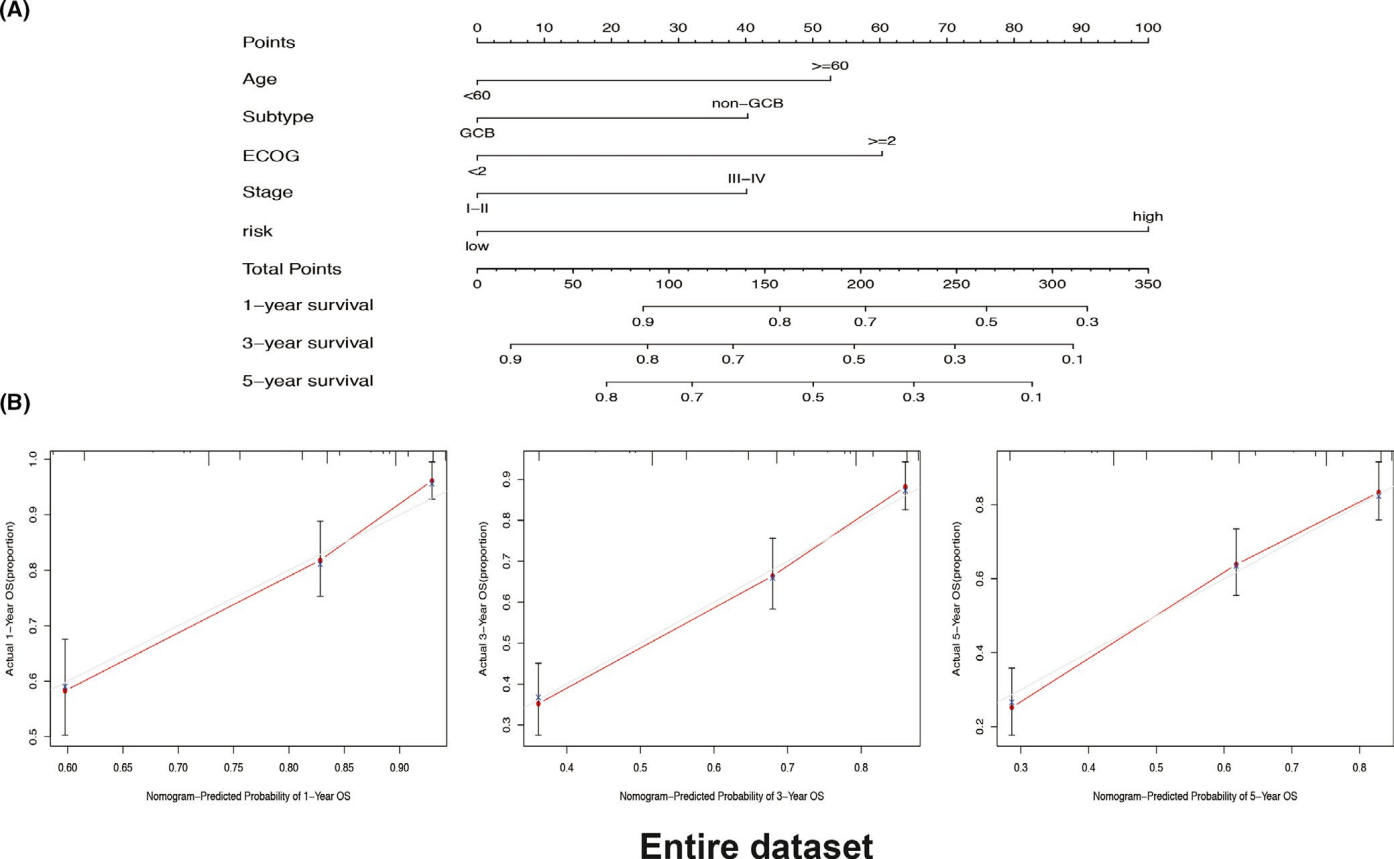
Construction and validation of a six‐RNA binding protein (RBP) signature‐based predictive nomogram in the entire dataset (*N* = 388) of GSE10846. (A) The six‐RBP signature‐based nomogram combining clinical risk factors for predicting 1‐, 3‐, and 5‐year survival of diffuse large B‐cell lymphoma patients. (B) Calibration curves of the nomogram for predicting 1‐, 3‐, and 5‐year survival

A prognostic nomogram combining six‐RBP signature and OS‐related clinical factors was constructed to predict the probability of 1‐, 3‐, ‐and 5‐year OS in DLBCL patients (patients lacking complete clinical information were excluded) (Figure 6A). Patients with higher score indicated a poor outcome and six‐RBP signature contributed most to the survival. Concordance index (C‐index) of nomogram was 0.74 (95% CI = 0.703–0.777) for the entire dataset and 0.774 (95% CI = 0.727–0.821) for the primary dataset (Figure S3A). Calibration plots showed a similar performance for predicting 1‐, 3‐, and 5‐year survival as compared to the ideal model in entire (Figure 6B) and primary datasets (Figure S3B).

**FIGURE 7:**
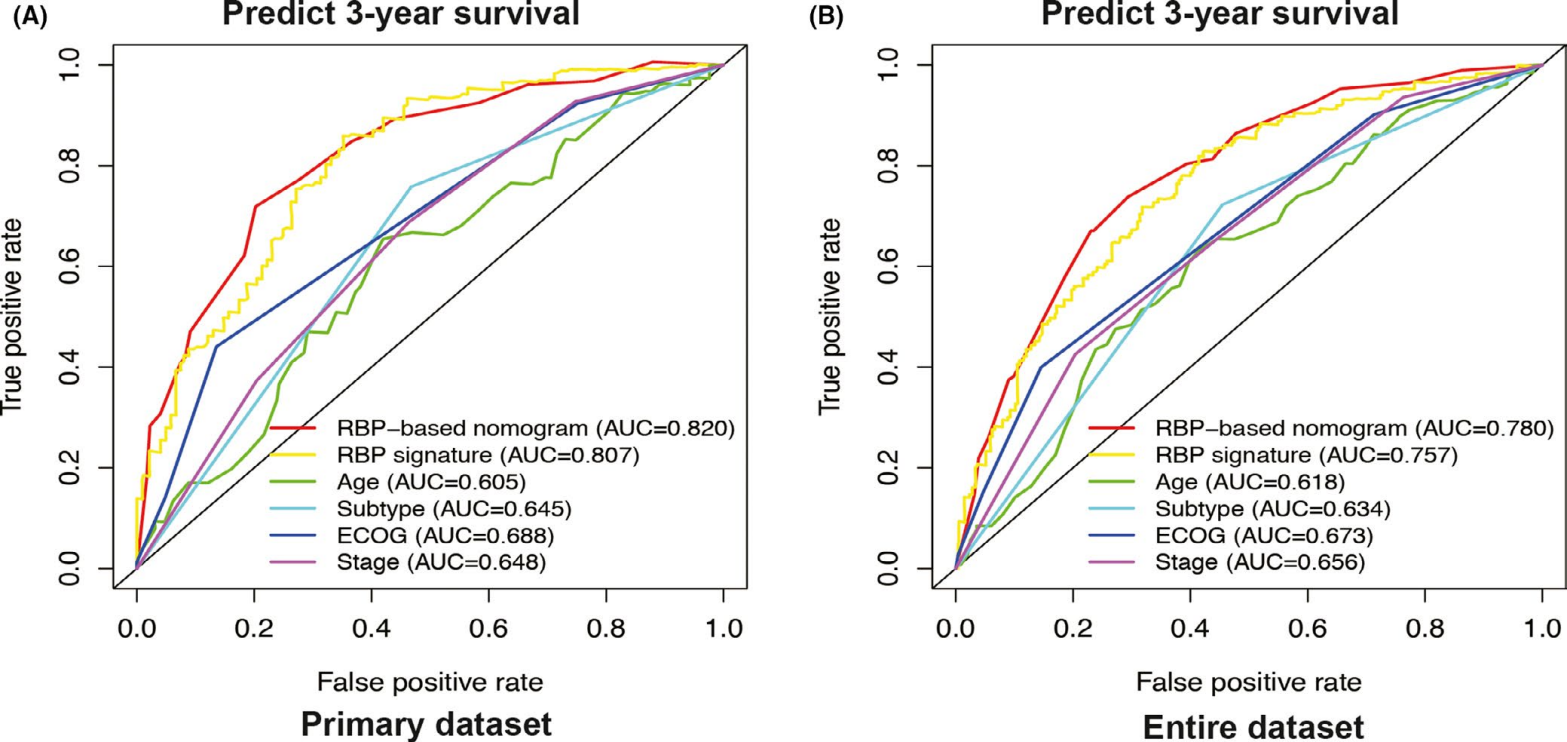
Estimate the prognostic accuracy of the six‐RNA binding protein (RBP) signature‐based nomogram to predict 3‐year survival in diffuse large B‐cell lymphoma (DLBCL) patients. Time‐dependent receiver operating characteristic curve analysis for clinical risk factors only, the six‐RBP signature, and six‐RBP signature combined with clinical risk factors for prediction 3‐year survival of DLBCL patients in the primary dataset (*N* = 199) and entire dataset (*N* = 388) of GSE10846

To compare the predictive power of compound nomogram and six‐RBP signature or clinical risk factors separately, we performed time‐dependent ROC curves analysis. For 3‐year survival prediction, AUC values of compound nomogram in the primary set (Figure 7A) and entire set (Figure 7B) were 0.820 and 0.780, respectively, which showed an obviously better predictive performance when comparing to the six‐RBP signature or clinical risk factors only. Similar performance was also observed in predicting 5‐year survival (Figure S4A,B). To sum up, our six‐RBP signature‐based nomogram had high prognostic predictive efficiency for DLBCL patients.

## 4 DISCUSSION

The significant heterogeneity of DLBCL is still remained challenging for clinicians in developing individualized treatments, patients with poor outcomes undoubtedly will suffer from great financial, health, and psychological problems.^1^ Therefore, there is an urgent need for methods to better predict the prognosis of DLBCL patients and provide more effective interventions according to prediction results. RBPs regulatory networks mainly impact cancer development by altering various cancer‐associated downstream targets.^7^ Once these networks were disturbed, they may cooperate with other primary carcinogenic hits to accelerate progression and increase the aggressiveness of tumor.^10^, ^13^ Recently increasing studies have demonstrated the important roles of RBP in hematological malignancy. *hnRNP K* had been identified as a bona fide oncogene and mitigating hnRNP K‐mediated c‐Myc activation could be a novel strategy for DLBCL patients.^8^ Recurrent mutations in *SRSF2* globally affected the RNA binding and splicing process, such as the hnRNP family of protein, resulting in poor clinical outcomes in myelodysplastic syndromes.^12^ Considering the importance of RBPs regulatory networks in cancer progression and hematological malignancy, it is essential to clarify the association between RBP and DLBCL and to develop a RBPs‐related risk assessment system to predict the prognosis of DLBCL patients.

In this study, we primarily focused on the genomic pathogenesis of the RBPs in association with the development of DLBCL and patients' OS. Further bioinformatic analysis helped to elucidate the RBPs regulatory networks, as well as to identify the biological functions of these RBPs‐associated genes. We found that six RBPs, including *CMSS1*, *MAEL*, *THOC5*, *PSIP1*, *SNIP1*, and *ZCCHC7*, are significantly related to OS of DLBCL patients, which could potentially be served as prognostic biomarkers. DLBCL patients with high‐risk score had a poor prognosis than those with low‐risk score. ROC curve analysis of the six‐RBP signature showed a high AUC value in predicting 3‐ and 5‐year survival, respectively (0.798 and 0.744 in primary set, 0.735 and 0.711 in the entire set), confirming the reliability of gene signature. More importantly, we performed multivariate CPHR analysis and demonstrated that the six‐RBP signature can be used as a novel independent indicator for the prognosis of DLBCL patients.

RNA binding protein have the ability to bind and regulate a myriad of transcripts. Among the identified six RBP, THOC5, SNIP1, PSIP1, and ZCCHC7 were served as translation regulators. THOC5 is involved in the regulation of transcription factor expression and the processing and transport of mRNA, which has an important role in hematopoietic stem cells for survival.^14^, ^15^, ^16^, ^17^ THOC5 couples macrophage colony‐stimulating factor receptor signaling and contributes to macrophage and monocyte differentiation, suggesting the potential roles in the development of lymphoma.^18^, ^19^, ^20^ Moreover, THOC5 phosphorylation is elevated in stem cells of CML patients, which may represent a novel strategy for CML therapy.^15^ SNIP1 enhances c‐MYC transcription by regulating c‐MYC stability and is overexpressed in many tumors.^21^, ^22^ Dysregulation of *c‐MYC* is essential in the pathogenesis of a number of B‐cell lymphomas, including DLBCL.^23^ SNIP1 also plays an important role in DNA damage response, cell viability, and proliferation during tumorigenesis.^24^ The SNIP1 expression in DLBCL has not been directly addressed, future studies will help to clarify the association between SNIP1 and DLBCL. Studies also demonstrated an important role of PSIP1 in tumorigenicity by regulating the transcription of genes that control the cell cycle and tumor metastasis.^25^, ^26^, ^27^ PSIP1 was also confirmed to be the target gene of miR‐155 in B‐cell lymphoma.^28^ Of note, miR‐155 was frequently upregulated in B‐cell lymphoproliferative diseases (such as aggressive activated B‐cell‐like subtype of DLBCL), indicating the important roles of PSIP1 in DLBCL.^29^ ZCCHC7 was found to have a putative role in human B cell development in adult bone marrow.^30^ Downregulation of ZCCHC7 was correlated with acute lymphoblastic leukemia (ALL) children at a high risk to relapse,^31^ similar to our data that low expression level of ZCCHC7 associated with poor outcome. Of note, recent studies reported that ZCCHC7 was the partner gene of MYC rearrangements in DLBCL.^32^ *MAEL* is a novel cancer/testis‐associated gene, which is not only aberrantly expressed in various cancer tissues but also characterized as a tumor‐promoting factor through activating Akt and regulating the phosphorylation of nuclear factor kappa B.^33^, ^34^ The functions of CMSS1 were seldom studied; however, the upregulation of CMSS1 was an observer in immortalized cells, cancer cells, and non‐small‐cell lung cancer tissues.^35^ Furthermore, GSEA analysis identified six‐RBP signature correlated signaling pathway and revealed that 17 pathways were enriched between high‐ versus low‐risk group, including the regulation of autophagy, CML, NOTCH signaling pathway, and B cell receptor signaling pathway. We suggested that the expression of six‐RBP might exert regulatory roles in these pathways. Of interest, the activation of autophagy was associated with high‐risk patients who had poor outcomes. Inhibition of autophagy might be the potential therapeutic target for DLBCL patients. Growing evidence supports that enhanced autophagy protects tumor cells from adverse conditions such as hypoxia and radiotherapy.^36^ Autophagy inhibition may be beneficial to patients with lymphoma.^37^, ^38^ Recently, Gayle et al provide evidence that the disruption of lysosomal homeostasis and inhibition of the autophagy flux could be a novel approach to treat B‐cell non‐Hodgkin lymphoma.^39^ NOTCH signaling pathway has been reported aberrantly activated in hematological malignancies, such as DLBCL and T‐cell ALL.^2^, ^5^, ^40^ Moreover, NOTCH pathway mutations significantly altered downstream target genes, emphasizing the roles of this pathway in DLBCL.^5^ Our analysis provided insight into the underlying mechanisms in the occurrence and development of DLBCL, which laid the foundation for further basic studies and might benefit from exploring novel intervention strategies.

We further constructed a compound prognostic nomogram combining RBP‐based gene signature with clinical factors. The nomogram model (AUC = 0.820 in primary set, AUC = 0.780 in the entire set) was superior to the RBP‐based gene signature and the clinical factors. Our newly developed model improved the prognostic predictive efficiency, which could be used to individualize the survival probability for patients and has the potential translation into clinical practice in the future.

There were some potential limitations in our study. Although our research is based on massive cohorts from the GEO databases to establish and validate RBPs‐related prognostic model, the present study nevertheless features a retrospective design. Thus, a well‐designed, prospective, international, multicenter clinical trial is needed to confirm our findings in the future. GSEA analysis will obtain different results if using different gene signatures on the basis of risk score for classification, the biological significance of identified pathways needs more studies to verify. Besides, more efforts should be channeled to investigate the molecular mechanism of the identified RBPs‐related genes during DLBCL progression.

In summary, we constructed for the first time a RBPs‐related risk prediction model, which had great potential on its application to predict DLBCL patients' survival. The present study also provides a potential biomarker for DLBCL screening and diagnosis, facilitating patient counseling and decision making. However, more studies are warranted to illuminate the contribution to the prognosis of DLBCL.

## CONFLICT OF INTEREST

All authors declare no conflict of interest.

## ETHICAL APPROVAL

This study does not contain any studies with human participants or animals performed by any of the authors, all analyses were based on public GEO databases.

## AUTHOR CONTRIBUTIONS

Yizi He designed the study and revised the manuscript; Yongzhi Xie, Ximei Luo, Haiqing He, and Tao Pan collected, arranged, and analyzed the data; Yongzhi Xie provides the first draft of the manuscript. All authors reviewed and approved the final manuscript.

## Supporting information

Fig S1

Fig S2

Fig S3

Fig S4

Table S1

Supplementary Material

## Data Availability

The datasets analyzed in this study can be found in the GEO database (https://www.ncbi.nlm.nih.gov/geo).
