# Supplementary figures and images for: Identification of an individualized RNA binding protein‐based prognostic signature for diffuse large B‐cell lymphoma
Source: Cancer Med. 2021 Mar 21;10(8):2703–13. doi: 10.1002/cam4.3859 (PMC8026940; doi:10.1002/cam4.3859)

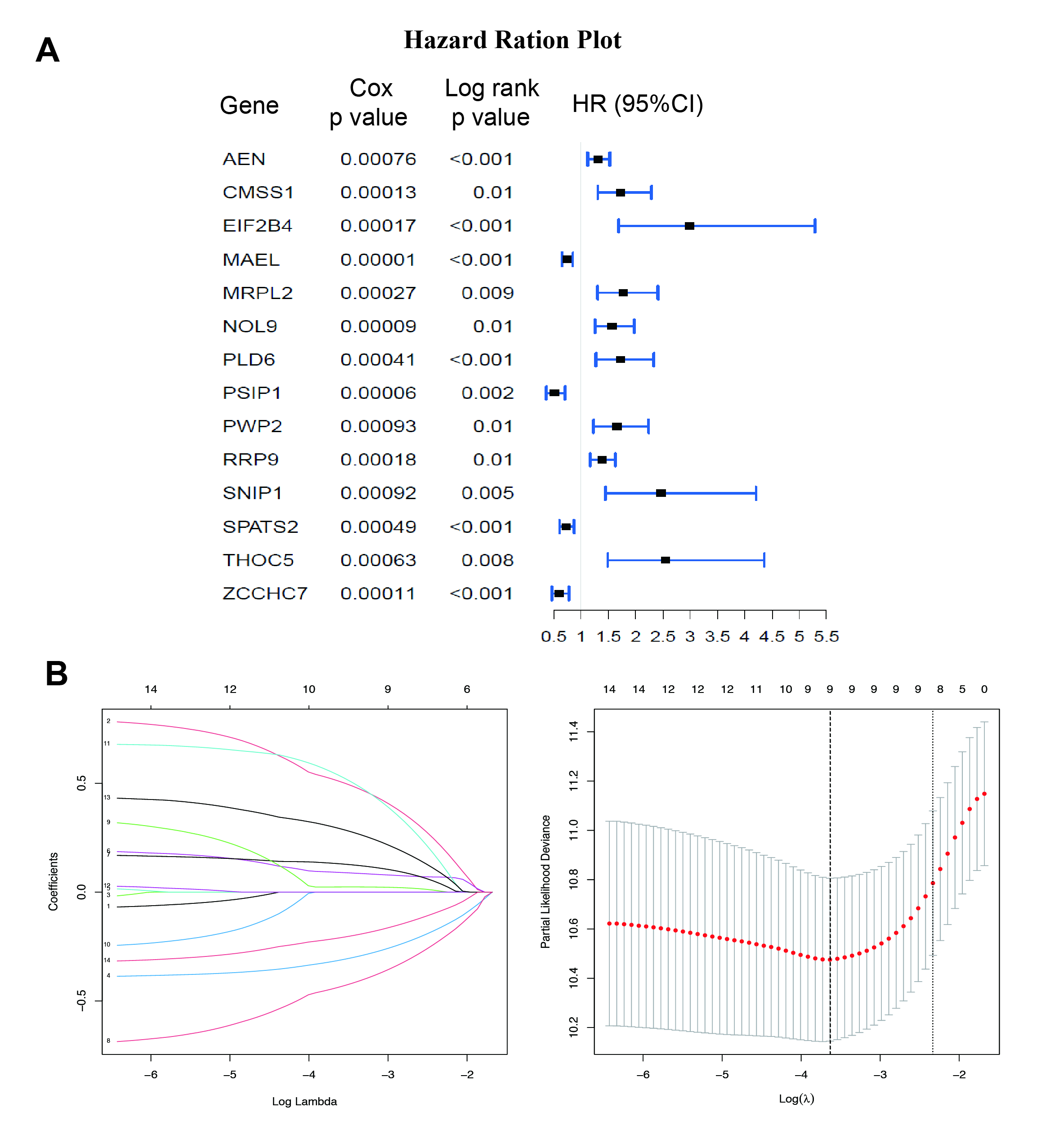

Supplement: Supplementary file 1 — Fig S1 [file CAM4-10-2703-s004.tif]

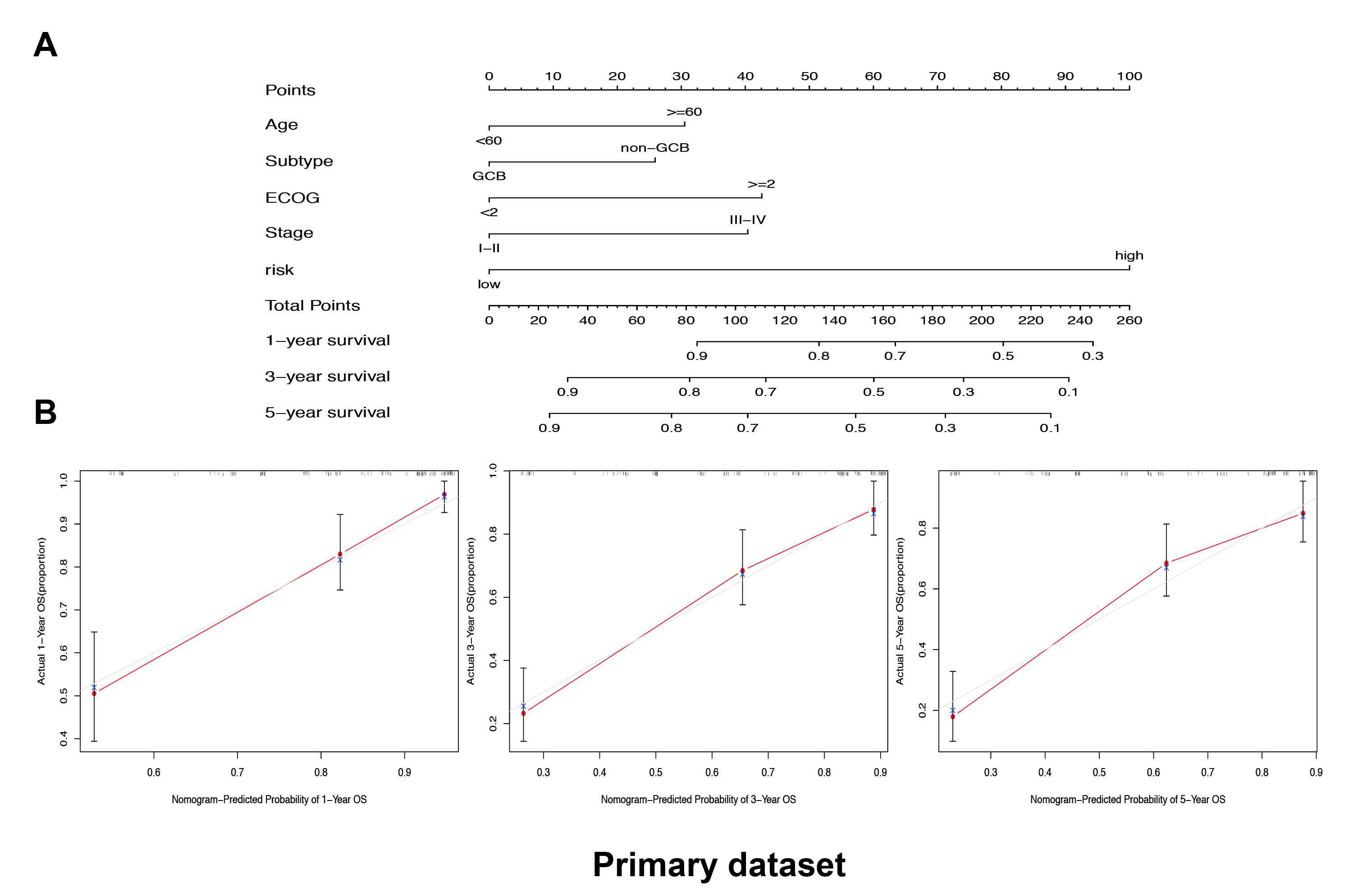

Supplement: Supplementary file 2 — Fig S2 [file CAM4-10-2703-s005.tif]

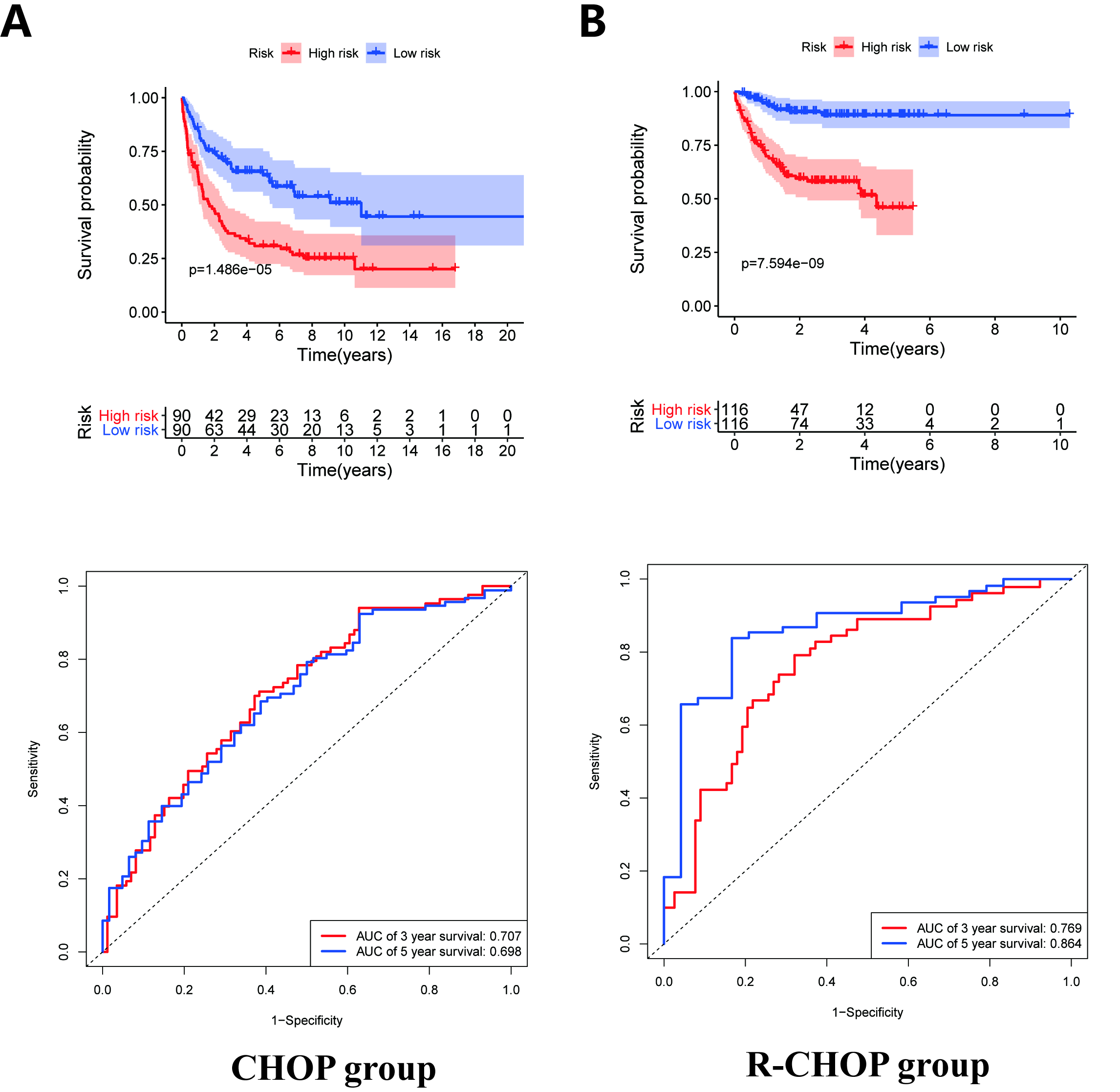

Supplement: Supplementary file 3 — Fig S3 [file CAM4-10-2703-s003.tif]

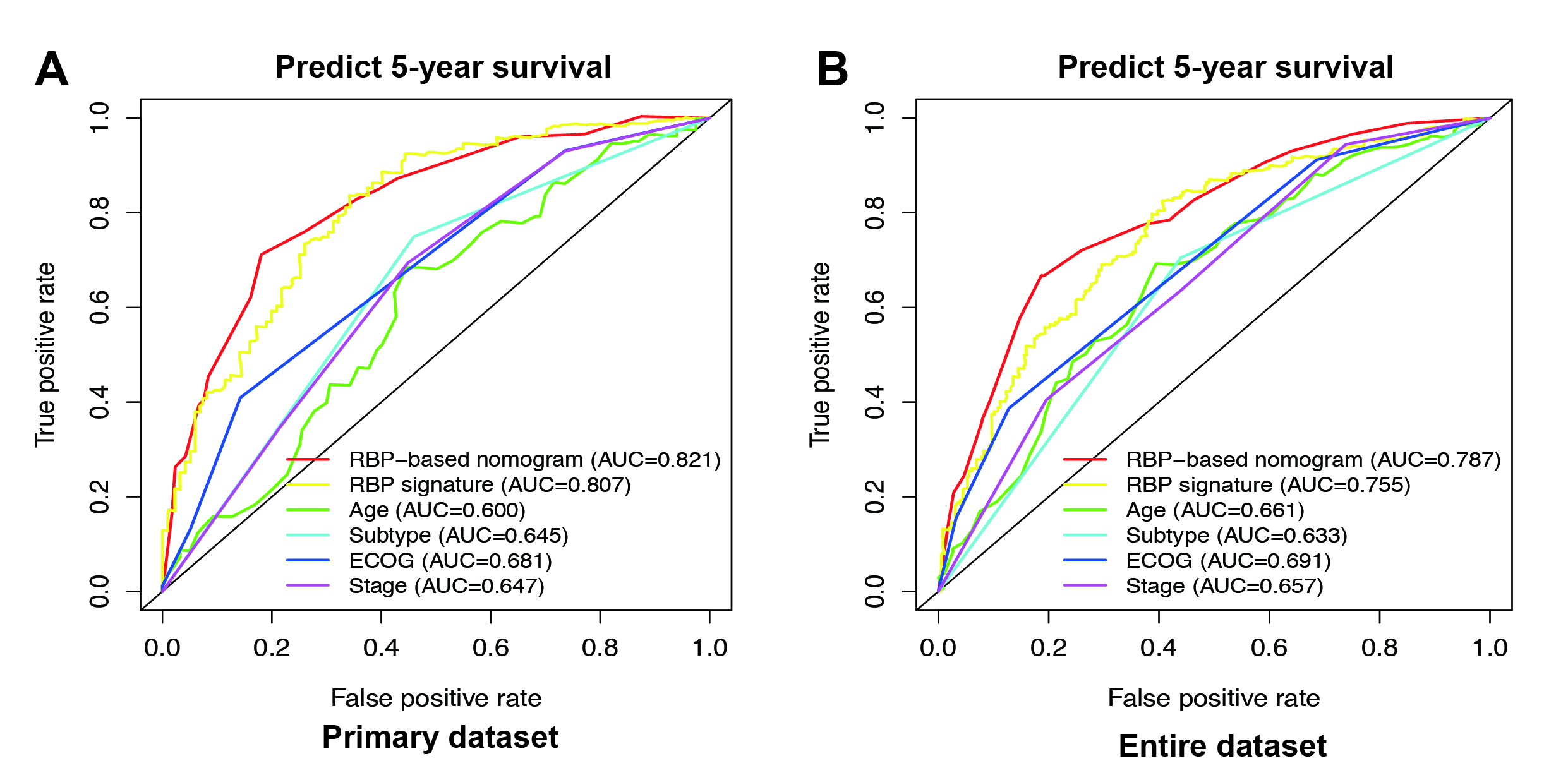

Supplement: Supplementary file 4 — Fig S4 [file CAM4-10-2703-s006.tif]
