# Supplementary material for: Identification of an individualized RNA binding protein‐based prognostic signature for diffuse large B‐cell lymphoma
Source: Cancer Med. 2021 Mar 21;10(8):2703–13. doi: 10.1002/cam4.3859 (PMC8026940; doi:10.1002/cam4.3859)
Supplement: Supplementary file 5 — Table S1 [file CAM4-10-2703-s001.docx]

**Supplementary Table 1** Gene set enrichment analysis between high- vs low-risk score group in GSE10846 dataset.

| KEGG pathway | NES | P value |
| --- | --- | --- |
| KEGG_REGULATION_OF_AUTOPHAGY | 1.507065 | 0.038462 |
| KEGG_FC_GAMMA_R_MEDIATED_PHAGOCYTOSIS | -1.78784 | 0.023392 |
| KEGG_RENAL_CELL_CARCINOMA | -1.78478 | 0.010081 |
| KEGG_SMALL_CELL_LUNG_CANCER | -1.76536 | 0.017208 |
| KEGG_CHRONIC_MYELOID_LEUKEMIA | -1.75776 | 0.037951 |
| KEGG_COLORECTAL_CANCER | -1.74377 | 0.029014 |
| KEGG_ENDOCYTOSIS | -1.74157 | 0.005693 |
| KEGG_N_GLYCAN_BIOSYNTHESIS | -1.70461 | 0.039526 |
| KEGG_ADHERENS_JUNCTION | -1.69295 | 0.028807 |
| KEGG_ERBB_SIGNALING_PATHWAY | -1.67843 | 0.007984 |
| KEGG_BIOSYNTHESIS_OF_UNSATURATED_FATTY_ACIDS | -1.63887 | 0.021484 |
| KEGG_B_CELL_RECEPTOR_SIGNALING_PATHWAY | -1.63738 | 0.049618 |
| KEGG_NOTCH_SIGNALING_PATHWAY | -1.59273 | 0.026639 |
| KEGG_FOCAL_ADHESION | -1.56651 | 0.034908 |
| KEGG_ADIPOCYTOKINE_SIGNALING_PATHWAY | -1.52577 | 0.018282 |
| KEGG_INSULIN_SIGNALING_PATHWAY | -1.52467 | 0.015414 |
| KEGG_TYPE_II_DIABETES_MELLITUS | -1.4807 | 0.023904 |

NES, normalized enrichment score
