# Supplementary material for: Identification of an individualized RNA binding protein‐based prognostic signature for diffuse large B‐cell lymphoma
Source: Cancer Med. 2021 Mar 21;10(8):2703–13. doi: 10.1002/cam4.3859 (PMC8026940; doi:10.1002/cam4.3859)
Supplement: Supplementary file 6 — Supplementary Material [file CAM4-10-2703-s002.docx]

**Supplementary figure 1** Identification of RBP-related genes associated with prognosis in DLBCL. (A) Forest plot of 14 RBP-related genes with p <0.01 in both univariate Cox regression and Kaplan-Meier analysis. (B) The least absolute shrinkage and selection operator (LASSO) Cox regression analysis was performed to identify RBP-related genes closely related to the survival time of DLBCL patients.

**Supplementary figure 2** Validation of Six-RBP gene signature in patients with CHOP or R-CHOP-based regimens. The six-RBP gene signature-based Kaplan–Meier analysis for overall survival (OS) and time-dependent ROC curve analysis for predicting 3- and 5-year OS in CHOP group (A) and in R-CHOP group (B). CHOP: cyclophosphamide, doxorubicin, vincristine, and prednisone; R-CHOP: rituximab plus CHOP.

**Supplementary figure 3** Construction and validation of a six-RBP signature-based predictive nomogram in the primary dataset (N=199) of GSE10846. (A) The six-RBP signature-based nomogram combining clinical risk factors for predicting 1-, 3-, and 5-year survival of DLBCL patients. (B) Calibration curves of the nomogram for predicting 1-, 3-, and 5-year survival.

**Supplementary figure 4** Estimate the prognostic accuracy of the six-RBP signature-based nomogram to predict 5-year survival in DLBCL patients. Time-dependent ROC curve analysis for clinical risk factors only, the six-RBP signature, and six-RBP signature combined with clinical risk factors for prediction 5-year survival of DLBCL patients in the primary dataset (N=199) and entire dataset (N=388) of GSE10846.
